# The interaction between the soluble programmed death ligand-1 (sPD-L1) and PD-1^+^ regulator B cells mediates immunosuppression in triple-negative breast cancer

**DOI:** 10.3389/fimmu.2022.830606

**Published:** 2022-07-22

**Authors:** Xuejiao Li, Huan Du, Shenghua Zhan, Wenting Liu, Zhangyu Wang, Jing Lan, Longxiang PuYang, Yuqiu Wan, Qiuxia Qu, Sining Wang, Yang Yang, Qin Wang, Fang Xie

**Affiliations:** ^1^ School of Biology & Basic Medical Sciences, Medical College of Soochow University, Suzhou, China; ^2^ Department of Pathology, The First People’s Hospital of Lianyungang, Lianyungang, China; ^3^ Department of Pathology, Huashan Hospital, Fudan University, Shanghai, China; ^4^ Department of Pathology, The First Affiliated Hospital of Soochow University, Suzhou, China; ^5^ Department of General Surgery, The First Affiliated Hospital of Soochow University, Suzhou, China; ^6^ Department of General Surgery, Suzhou Kowloon Hospital, Shanghai Jiao Tong University School of Medicine, Suzhou, China; ^7^ Jiangsu Institute of Clinical Immunology, The First Affiliated Hospital of Soochow University, Suzhou, China

**Keywords:** sPD-L1, PD-1+ regulator B cells, regulator T cell, immunosuppression, the triple-negative breast cancer

## Abstract

Accumulating evidence suggests that regulatory B cells (Bregs) play important roles in inhibiting the immune response in tumors. Programmed death 1 (PD-1) and programmed death ligand 1 (PD-L1) are important molecules that maintain the balance of the immune response and immune tolerance. This study aims to evaluate the soluble form of PD-L1 and its function in inducing the differentiation of B lymphocytes, investigate the relationship between soluble PD-L1 (sPD-L1) and B-cell subsets, and explore the antitumor activity of T lymphocytes after PD-L1 blockade in coculture systems. In an effort to explore the role of sPD-L1 in human breast cancer etiology, we examined the levels of sPD-L1 and interleukin-10 (IL-10) in the serum of breast tumor patients and the proportions of B cells, PD-1^+^ B cells, Bregs, and PD-1^+^ Bregs in the peripheral blood of patients with breast tumors and assessed their relationship among sPD-L1, IL-10, and B-cell subsets. The levels of sPD-L1 and IL-10 in serum were found to be significantly higher in invasive breast cancer (IBCa) patients than in breast fibroadenoma (FIBma) patients. Meanwhile, the proportions and absolute numbers of Bregs and PD-1^+^ Bregs in the peripheral blood of IBCa patients were significantly higher than those of FIBma patients. Notably, they were the highest in triple-negative breast cancer (TNBC) among other subtypes of IBCa. Positive correlations of sPD-L1 and IL-10, IL-10 and PD-1^+^ Bregs, and also sPD-L1 and PD-1^+^ Bregs were observed in IBCa. We further demonstrated that sPD-L1 could induce Breg differentiation, IL-10 secretion, and IL-10 mRNA expression in a dose-dependent manner *in vitro*. Finally, the induction of regulatory T cells (T_regs_) by Bregs was further shown to suppress the antitumor response and that PD-L1 blockade therapies could promote the apoptosis of tumor cells. Together, these results indicated that sPD-L1 could mediate the differentiation of Bregs, expand CD4^+^ T_regs_ and weaken the antitumor activity of CD4^+^ T cells. PD-L1/PD-1 blockade therapies might be a powerful therapeutic strategy for IBCa patients, particularly for TNBC patients with high level of PD-1^+^ Bregs.

## Introduction

Invasive breast cancer (IBCa) is the most common cancer that threatens women’s health worldwide (1). Triple-negative breast cancer (TNBC), accounting for approximately 10%–15% of all diagnosed breast cancers, is associated with high tumor heterogeneity, low differentiation, rapid proliferation, early clinical recurrence and metastasis, and poor clinical outcomes (2). Our previous study showed that MDA-MB231 cells from TNBC patient expressed a higher level of programmed death ligand 1 (PD-L1), which induced regulatory B cells (Bregs) under coculture conditions and played an important role in regulating antitumor immune suppression. Dirix et al. and Mittendorf et al. showed that approximately 20% of TNBC tumor tissues express PD-L1, and the majority of these TNBC tumors were grade 3 (3, 4). Our previous study also found PD-L1 expression in 83.6% (112 of 134) of IBCa cases evaluated, which was significantly associated with Tumor Node Metastasis (TNM) staging (5).

Recently, immunotherapy, such as PD-L1/programmed death 1 (PD-1) blockade, has revolutionized the management of multiple solid tumors. Early data have shown promising outcomes in small numbers of advanced TNBC patients treated with PD-L1/PD-1 antagonists, such as atezolizumab and pembrolizumab (6). Immune checkpoint blockade therapies have brought bright prospects for patients with metastatic TNBC (7).

PD-L1, known as CD274 or B7-H1, belongs to the B7 family and is expressed on B lymphocytes, T lymphocytes, monocytes, antigen-presenting cells (APCs), and epithelial cells (8). Furthermore, PD-L1 is expressed in a series of human malignancies and has been reported to play a critical role in suppressing anticancer immunity by binding to the receptor PD-1 (9). Structurally, PD-L1 contains immunoglobulin V (IgV)– and immunoglobulin C (IgC)–like extracellular domains, a transmembrane domain, and a short cytoplasmic domain (10), whereas receptor PD-1 comprises an IgV-like extracellular domain, a transmembrane domain, and a cytoplasmic domain that harbors a cytoplasmic immunoreceptor tyrosine–based inhibitory motif (ITIM) and an immunoreceptor tyrosine–based switch motif (ITSM) (11). Interaction between the extracellular domains of PD-L1 and PD-1 can induce a conformational change in PD-1 that leads to phosphorylation of ITIM and ITSM to reduce the strength of the TCR signal (10). Moreover, the interaction of PD-L1 and PD-1 can transduce inhibitory signals to effector T cells (T_effs_) (12).

As one of the B7 family members, the soluble form of PD-L1 was found and initially described in autoimmune disorders, which were thought to be produced by immune cells upon stimulation with proinflammatory cytokines (13). Recent publications have described elevated levels of soluble PD-L1 (sPD-L1) in patients with some human cancers, which could be used as biomarkers reflecting clinical status, treatment response, and disease outcome (14–16). Interestingly, Li et al. and Yazdanpanah et al. found that sPD-L1 plasma levels were significantly increased in TNBC patients and could be used to predict the treatment response to immunotherapy (17, 18). However, studies elucidating the role of sPD-L1 in regulating the function of Bregs in immune suppression are rare.

Here, to investigate sPD-L1 as a soluble form of PD-L1 in immune suppression, we retrospectively evaluated the level of sPD-L1 in the blood plasma of IBCa patients and analyzed the relationship of sPD-L1 expression with the presence of Bregs; clinical characteristics including TNM stage, tumor grade, lymph node infiltration; and the presence of metastases as well as the antitumor activity of T cells in breast cancer and lymphocyte coculture systems.

In this study, the effect of sPD-L1 on regulating the differentiation and functions of B cells and their subsets was further explored, and it provides evidence for the future use of immune checkpoint blockade therapies in IBCa and TNBC patients.

## Materials and methods

### Reagents

The following monoclonal antibodies and reagents were used in this study: anti-human CD24-PE-cy7 (clone: eBioSN3), anti-human CD4-BV605 (clone: RPA-T4), anti-human CD25-BV421 (clone: M-A251), anti-human CD19-BV605 (clone: SJ25C1), and anti-human PD-1-FITC (clone: MIH4) were purchased from eBioscience (USA); anti-human PD-L1-PE (clone: 291.2A3), anti-human CD38-BV421 (clone: HIT2), and anti-human CD127-PE-cy7 (clone: A019D5) were purchased from BioLegend (USA); anti-human Foxp3-APC (clone: 3G3) and anti-human CD4 MicroBeads were purchased from Miltenyi Biotec (German); CellTrace™ CFSE Cell Proliferation Kit, Annexin V-APC/PI Apoptosis kit, Annexin V-APC/7-AAD Apoptosis kit, and Human IL-10 Uncoated ELISA kit were purchased from Invitrogen (USA); sPD-L1 ELISA kit and anti-human PD-L1 blocking antibody (clone: 2H11) were obtained from Suzhou Bright Scistar Biotechnology (China); Human B-cell enrichment kit (catalog: 19054) was purchased from STEMCELL Technologies (Canada); PrimeScript™ RT Master Mix and SYBR Premix Ex Taq™ were purchased from TaKaRa (Japan); lipopolysaccharide (LPS) was purchased from Sigma-Aldrich (USA); anti-CD3e monoclonal antibody was purchased from Glucan Biotech. (clone: OKT3; China); BD Matrigel™ matrix was purchased from BD Biosciences (USA); sPD-L1 was purchased from Biointron (China); and Transwell plates was purchased from Corning Incorporated (USA).

The following monoclonal antibodies were prepared in our laboratory: anti-human CD28 antibodies (clone: 18G8) (5) and agonist anti-human CD40 monoclonal antibody (clone: 5C11) (19).

### Patients

Peripheral blood cells and serum of IBCa and fibroadenoma (FIBma) patients were randomly collected from the archive of the First Affiliated Hospital of Soochow University. The samples were centrifuged at 1500 rpm for 10 min, the cell-free sera were stored at −20°C for enzyme-linked immunosorbent assay (ELISA), and the peripheral blood cells were analyzed for cell surface markers by flow cytometry.

The cases from 114 cases of IBCa and 26 cases of FIBma from the breast were analyzed in the present study. None of the patients with IBCa had received any kind of chemical or radiation therapy before surgery. The age of the IBCa patients ranged from 31 to 90 years, with a mean of 53.7 years. The histopathological diagnosis of breast tumors was made according to cellular morphological changes and tissue architecture by using previously established criteria. Fourteen of 114 IBCa cases were graded as well differentiated (12.3%), 73 of 114 were moderately differentiated (64.0%), and the rest (23.7%, 27 of 114) were poorly differentiated. The TNM stage of invasive carcinomas was also assessed following the WHO classification of breast tumors as previously reported (5). Carcinoma specimens were classified as stage T1 (36 of 114, 31.6%), T2 (48 of 114, 42.1%), T3 (19 of 114, 16.7%), and T4 (11 of 114, 9.6%). In addition, local lymph node metastasis occurred in 54 of 114 cases (47.4%) (**Supplemental Table 1**).

The expression levels of estrogen receptor (ER), progesterone receptor (PR), human epidermal growth factor receptor 2 (HER2), and Ki67 were scored according to the guidelines of the National Comprehensive Cancer Network of our previous report (5). Briefly, a case was considered positive for ER and PR if more than 15% of the tumor cells showed nuclear staining of ER, PR, or Ki67. Positivity for HER2 was scored as “++” when strong and complete membrane staining was present in more than 30% of invasive tumor cells. FIBma biopsies were taken from 26 symptom-free female subjects for which the median age was 50.0 (17~62) years old.

This study was approved by the ethics committee of the University of Soochow Hospitals National Health Service Trust. Patients and healthy volunteers were recruited after informed consent was obtained.

### Breast cancer cell lines and culture

The human breast cancer lines MDA-MB231 and MCF-7 were purchased from American Type Culture Collection (ATCC) and cultured in RPMI 1640 medium (HyClone, Logan, UT, USA) containing 10% heat-inactivated fetal bovine serum (FBS) (Biological Industries, Israel) and 1% penicillin/streptomycin. All breast cancer cell lines used in these studies were assessed by flow cytometry analysis for constitutive cell surface PD-L1 expression (**Supplemental Figure 1A**).

### Cell isolation from peripheral blood

CD4^+^ T cells were purified from the peripheral blood of healthy individuals using a CD4^+^ T-cell–positive isolation kit according to the manufacturer’s instructions (Miltenyi Biotec, Germany). To establish T_H_0 cell conditions following our previous report, 2 × 10^5^ CD4^+^ T cells per well in 96-well plates were stimulated with anti-CD3e monoclonal antibody (1 μg/ml) and anti-CD28 monoclonal antibody (1 μg/ml) for 72 h. On day 3, activated CD4^+^ T cells were collected for coculture experiments.

A human B-cell enrichment kit was used to purify CD19^+^ B cells from the peripheral blood of healthy individuals according to the manufacturer’s instructions (Miltenyi Biotec, Germany). A total of 2 × 10^5^ purified CD19^+^ B cells were stimulated with LPS (1 μg/ml), anti-CD40 monoclonal antibody (1 μg/ml, clone: 5C11), or sPD-L1 (10–1,000 ng/ml) for subsequent coculture experiments.

### Depletion of PD-L1 by block antibody

We used a blocking antibody against PD-L1 at different concentrations to block PD-L1 expression on MDA-MB231 cells (**Supplemental Figure 1B**) for 30 min. At 0.5 h after blocking, the cells were harvested and subjected to analysis of PD-L1 expression by flow cytometry. Each block experiment was performed in triplicate and repeated three times.

### Coculture systems

#### CD19^+^ B-cell and CD4^+^ T-cell coculture systems

CD19^+^ B-cell and CD4^+^ T-cell coculture systems were established to study the interactions between immune cells. CD19^+^ B cells (2 × 10^5^) stimulated with LPS (1 μg/ml) or sPD-L1 (1000 ng/ml) were collected and cultured with activated CD4^+^ T cells at a ratio of 1:1. On day 1 after coculturing, CD4^+^ T cells were collected and washed for surface or intracellular staining.

#### CD19^+^ B cell and breast cancer cell coculture systems

Two coculture systems were established to investigate a potential association between CD19^+^ B cells and the soluble form of PD-L1 from breast cancer cells. The direct coculture system (DR) was performed as previously reported. Briefly, 2 × 10^5^ CD19^+^ B cells from healthy individuals stimulated with LPS were collected and cultured with PD-L1^hi^ MDA-MB231 or PD-L1^lo^ MCF-7 cells directly with or without blocking anti–PD-L1 antibody at a ratio of 10:1.

The indirect coculture system (IDR) was performed in Transwell chambers coated with BD Matrigel™ matrix (3 mg/ml) (BD Biosciences, Becton Dickson Labware, Franklin Lakes, NJ). Breast cancer cell lines, either PD-L1^hi^ MDA-MB231 or PD-L1^lo^ MCF-7 cells with or without previously treated anti–PD-L1 blockade, were seeded at 2 × 10^4^ cells per upper chamber well in Transwell culture plates and cocultured with activated CD19^+^ B cells from healthy individuals at a ratio of 1:10. At 24 h after coculturing, CD19^+^ B cells from the lower chamber in Transwell culture plates were collected and washed for surface staining.

#### CD19^+^ B-cells, CD4^+^ T-cells, and breast cancer cell coculture systems

CD19^+^ B cell, CD4^+^ T cell, and breast cancer cell coculture experiments were employed to investigate the antitumor immune response to PD-L1 signal blockade. Breast cancer cell lines, either PD-L1^hi^ MDA-MB231 or PD-L1^lo^ MCF-7 cells with or without anti–PD-L1 blocking antibody treatment previously, were seeded in 96-well culture plates and cocultured with CD19^+^ B cells stimulated with sPD-L1 or LPS with or without activated CD4^+^ T cells at a ratio of 1:5:5. Each experiment was performed in triplicate and repeated three times.

### Flow cytometry analysis

#### Surface staining and flow cytometry

The isolated peripheral blood mononuclear cells (PBMCs) were washed in PBS containing 2.5% FBS and incubated with specific fluorochrome-conjugated antibodies for 30 min at 4°C, including CD19-BV605, CD38-BV421, CD24-PE-Cy7, and PD-1-FITC, to identify B-cell surface molecules and CD4-BV605, CD127-PE-Cy7, and CD25-BV421 to identify T-cell surface molecules. After being washed, the labeled cells were resuspended in 0.5 ml of cell staining buffer and analyzed with flow cytometry software (FlowJo version 10, USA). Anti-mouse IgG isotype controls were performed for each staining.

#### Intracellular staining and flow cytometry

The surface molecules of regulatory T cells (T_regs_) were also stained as described above using fluorochrome-conjugated antibodies against CD4-BV605 and CD25-BV421. For intracellular staining, washed cells were fixed with Foxp3 fixation/permeabilization solution and incubated with APC-conjugated anti-Foxp3 antibody in the dark for 30 min. Labeled cells were resuspended in 0.5 ml od cell staining buffer and analyzed with flow cytometry.

#### Apoptosis analysis

Apoptosis was measured using an apoptosis detection kit. Briefly, MDA-MB231 cells stained with carboxyfluorescein diacetate succinimidyl ester (CFSE) were seeded in 96-well plates and incubated with T cells or B cells as described above. Twenty-four hours later, the cells were collected and stained with Annexin V-APC/PI or Annexin V-APC/7AAD and detected with flow cytometry (CytomicsTM FC500, Beckman Coulter, USA). All flow cytometry data were analyzed with FlowJo software (version 10, USA).

### Enzyme-linked immunosorbent assay

The levels of sPD-L1 and interleukin-10 (IL-10) in the serum of patients were determined in duplicate wells using sPD-L1 ELISA kits (Suzhou Bright Scistar, China) and IL-10 ELISA kits (Invitrogen, USA) according to the manufacturer’s instructions.

### Quantitative real-time PCR

CD19^+^ B cells (2 × 10^5^) were treated with LPS, 5C11, or sPD-L1 for 24 h. Twenty-four hours later, CD19^+^ B cells were collected for RNA isolation and quantitative real-time PCR analysis.

Total RNA was extracted from cells using TRIzol reagent and transcribed to cDNA with a PrimeScript RT Master Mix kit (TaKaRa, Japan) following the manufacturer’s instructions. Quantitative real-time PCR analysis of IL-10 gene expression was performed with a SYBR Green I Master kit (TaKaRa, Japan) (CFX96 Touch™ Real-Time System, Bio-Rad, USA). All data were normalized using β-actin as an internal control. Gene expression was expressed as the fold change from the β-actin level, which was calculated as the 2^-ΔΔCt^ method. In addition, melting curve analysis was performed to assure the specificity of the PCR product in this experiment. The primer sequences used for β-actin and IL-10 are shown in **Supplemental Table 2**. All experiments were carried out in triplicate.

### Statistical analysis

All of the data are presented as the means ± standard error of the mean and were analyzed using unpaired two-tailed Student’s *t*-tests or ANOVA. Correlations were determined by the Pearson’s and Spearman’s methods. Statistical significance was defined as *P* < 0.05. All analyses were performed using SAS 9.2 (SAS Enterprise Guide 3.0, Cary, NC).

## Results

### Relationship among B-cell subsets and IL-10 and sPD-L1 levels in breast cancer

**Figure 1:**
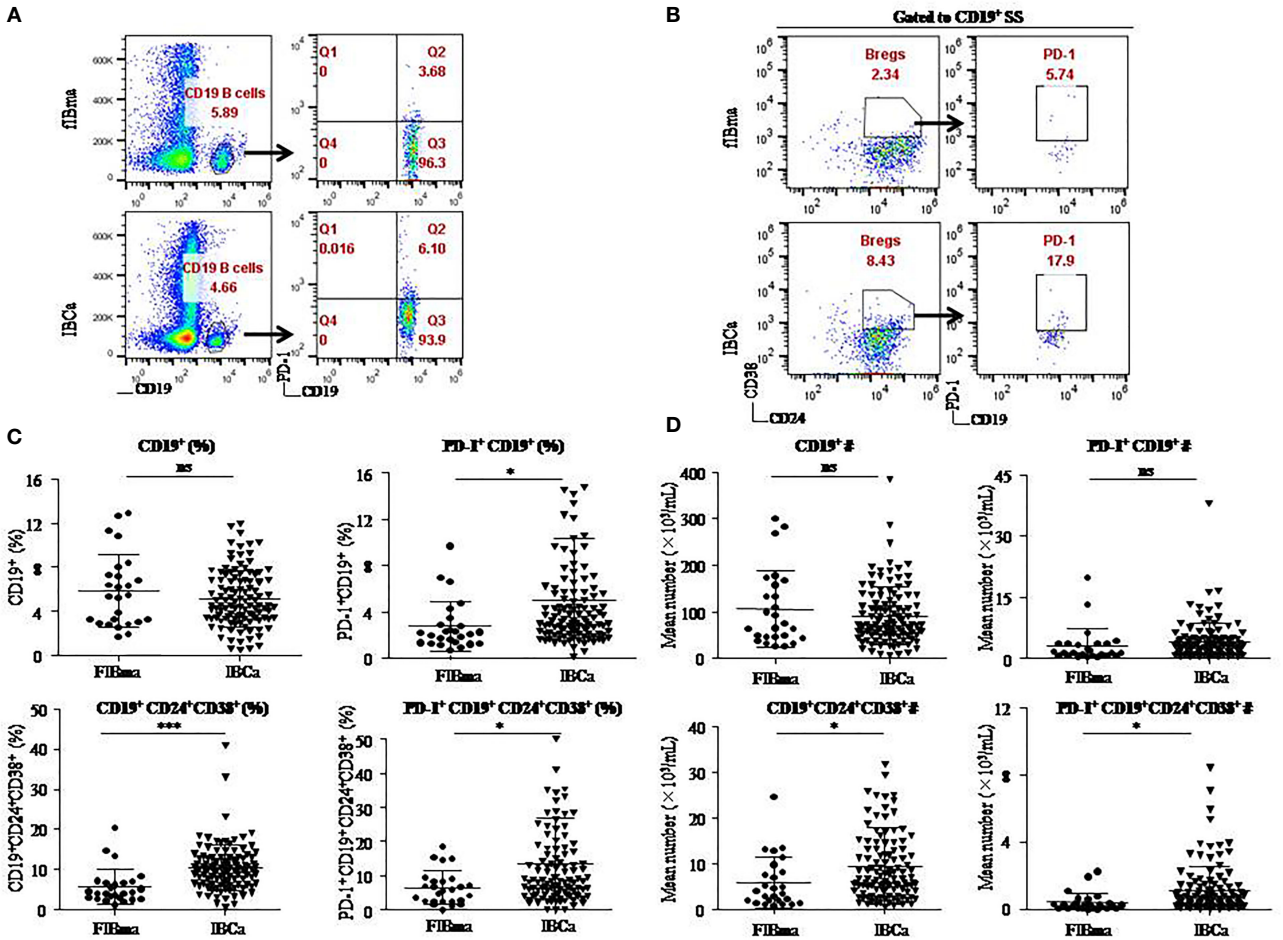
CD19^+^CD24^+^CD38^+^ B lymphocytes predominated in PBMCs of IBCa patients. The percentage and the absolute number of CD19^+^ B-cell subsets were measured in IBCa patients (n = 114) and FIBma patients (n = 26). PBMCs were isolated from peripheral blood and stained with CD19-BV605, CD24-PE-Cy7, CD38-BV421, and PD-1-FITC for flow cytometry, as described in “Materials and methods”. Representative flow cytometry dot plot of PD-1 expression in total CD19^+^ B cells **(A)** and CD24, CD38, and PD-1 expression in CD19^+^ B cells or CD19^+^CD24^+^CD38^+^ B cells **(B)**. Scatter plots showing the percentage of CD19^+^ B-cell subsets in FIBma patients and IBCa patients. A significant increase in the frequency of PD-1^+^CD19^+^B cells, CD19^+^CD24^+^CD38^+^ B cells, and PD-1^+^CD19^+^CD24^+^CD38^+^ B cells was observed in IBCa patients compared to FIBma patients (*P* = 0.035, *P* = 0.0001, *P* = 0.010, respectively) **(C)**. The absolute number of CD19^+^ B-cell subsets in FIBma patients and IBCa patients. The absolute numbers of CD19^+^CD24^+^CD38^+^ B cells and PD-1^+^CD19^+^CD24^+^CD38^+^ B cells were significantly higher in IBCa patients than in FIBma patients (*P* = 0.038 and *P* = 0.017, respectively) **(D)**. All values show the means ± SEM. Data were analyzed by unpaired Student’s *t*-test. ns, not statistically significant. *P < 0.05 and ***P < 0.001.

To evaluate the role of CD19^+^ B-cell subsets and PD-1 expression in the peripheral blood of patients (**Figures 1A, B**), the frequency and absolute number were measured in total CD19^+^ B cells in IBCa and FIBma. The percentages of PD-1^+^CD19^+^ B cells, CD19^+^CD24^+^CD38^+^ B cells, and PD-1^+^CD19^+^CD24^+^CD38^+^ B cells were significantly increased in patients with IBCa compared to patients with FIBma (*P* = 0.035, *P* = 0.0001, and *P* = 0.010, respectively) (**Figure 1C**). The average frequency of CD19^+^ B cells in IBCa patients was lower than that in FIBma patients, but no significant difference was observed (**Figure 1C**). The absolute numbers of both CD19^+^CD24^+^CD38^+^ B cells and PD-1^+^CD19^+^CD24^+^CD38^+^ B cells were significantly increased in IBCa compared to FIBma (*P* = 0.038, *P* = 0.017, respectively) (**Figure 1D**). No significant difference was observed in the absolute numbers of CD19^+^ B cells and PD-1^+^CD19^+^ B cells between IBCa and FIBma (*P* = 0.353) (**Figure 1D**). Further analysis of the relationships between the percentages and absolute numbers of CD19^+^ B-cell subsets and the histopathological characteristics of IBCa demonstrated that the percentages and absolute numbers of CD19^+^ B cells, PD-1^+^CD19^+^ B cells, CD19^+^CD24^+^CD38^+^ B cells, and PD-1^+^CD19^+^CD24^+^CD38^+^ B cells had no significant association with histological grade, lymph node metastasis, TNM stage, tumor size, ER status, or PR status (*P* > 0.05 for all; **Tables 1** **-** **4** **)**, except that the percentage of PD-1^+^CD19^+^ B cells was higher in IBCa with a small tumor size (6.06 ± 6.66%) than in IBCa with a large tumor size (3.86 ± 2.75%) (*P* = 0.028; **Table 1**). Although no statistically significant differences were observed, the percentages of PD-1^+^CD19^+^ B cells, CD19^+^CD24^+^CD38^+^ B cells, and PD-1^+^CD19^+^CD24^+^CD38^+^ B cells were increased in TNBC compared to other subtypes of IBCa (**Tables 1**, **2**).

**Table 1:** Relationship between the proportion of B cells and its subset in PBMCs and the clinicopathological parameters of IBCa patients.

Variables	All cases	B cells (%)	*P* value	PD-1^+^ B cells (%)	*P* value
IBCa	114				
Grade					
G1	14	5.05±3.25	0.619	4.78±5.00	0.778
G2	73	4.99±2.18		5.32±6.03	
G3	27	5.54±3.00		4.49±3.23	
LN metastasis					
NO	60	5.13±2.81	0.987	5.27±6.42	0.651
YES	54	5.12±2.19		4.81±3.85	
TNM					
T1	37	4.80±2.59	0.519	6.39±7.87	0.193
T2	46	5.43±2.73		4.54±3.46	
T3~T4	31	5.04±2.08		4.28±3.72	
ER					
Negative	40	5.08±2.63	0.878	5.86±7.24	0.240
Positive	74	5.15±2.48		4.62±3.95	
PR					
Negative	60	5.08±2.71	0.844	5.22±6.08	0.736
Positive	54	5.18±2.32		4.88±4.43	
HER-2					
Negative	74	5.24±2.50	0.639	5.00±5.99	0.869
Positive	40	4.92±2.58		5.17±3.94	
Age					
<49y	46	5.13±2.64	0.999	5.74±6.86	0.263
≥49y	68	5.13±2.46		4.59±4.00	
Tumor size					
<3cm	62	4.78±2.40	0.107	6.06±6.66	0.028
≥3cm	52	5.54±2.62		3.86±2.75	
Subtype					
Luminal A	17	5.72±2.46	0.337	4.95±5.12	0.340
Luminal B	58	4.98±2.46		4.48±3.57	
HER2-positive	20	4.50±2.23		4.91±4.17	
TNBC	19	5.72±2.85		7.06±9.28	

The meaning of “variables” is features of invasive breast cancer (IBCa). The meaning of “All cases” is the cases of IBCa. The meaning of “B cells (%)” is the percentage of CD19+ B cells in the peripheral blood mononuclear cells (PBMCs) of IBCa patients. The meaning of “PD-1+ B cells (%)” is the percentage of PD-1+ CD19+ B cells in PBMCs of IBCa patients. The meaning of “P value” is the results of statistical analysis.

**Table 2:** Relationship between the proportion of Bregs and its subset in PBMCs and the clinicopathological parameters of IBCa patients.

Variables	All cases	Bregs (%)	*P* value	PD-1^+^ Bregs (%)	*P* value
IBCa	114				
Grade					
G1	14	10.18±3.13	0.842	13.70±12.41	0.671
G2	73	10.65±6.38		14.16±15.11	
G3	27	9.95±4.08		11.43±8.86	
LN metastasis					
NO	60	10.90±6.31	0.344	12.77±13.40	0.570
YES	54	9.91±4.60		14.22±13.72	
TNM					
T1	37	11.46±5.43	0.405	15.90±15.62	0.426
T2	46	9.98±6.10		12.25±12.36	
T3~T4	31	9.90±4.77		12.47±12.58	
ER					
Negative	40	11.03±7.44	0.401	15.35±16.32	0.275
Positive	74	10.11±4.25		12.44±11.71	
PR					
Negative	60	10.99±6.43	0.262	14.03±14.31	0.639
Positive	54	9.81±4.39		12.83±12.66	
HER-2					
Negative	74	10.46±6.09	0.925	13.18±13.02	0.768
Positive	40	10.36±4.50		13.97±14.52	
Age					
<49y	46	11.50±6.80	0.091	12.61±12.76	0.581
≥49y	68	9.70±4.46		14.04±14.06	
Tumor size					
<3cm	62	10.84±5.20	0.340	15.44±14.62	0.088
≥3cm	52	9.94±5.98		11.10±11.76	
Subtype					
Luminal A	17	10.29±4.28	0.230	11.26±12.01	0.600
Luminal B	58	9.96±4.29		12.74±11.61	
HER2-positive	20	9.64±4.55		14.18±17.13	
TNBC	19	12.82±9.19		16.88±15.24	

**Table 3:** Relationship between the absolute number of B cells and its subset in PBMCs and the clinicopathological parameters of IBCa patients.

Variables	All cases	B cells (×10^3^)	*P* value	PD-1^+^ B cells (×10^3^)	*P* value
IBCa	114				
Grade					
G1	14	107.16±101.66	0.479	5.75±9.83	0.361
G2	73	86.88±47.43		3.87±3.63	
G3	27	96.24±68.88		3.80±2.80	
LN metastasis					
NO	60	95.34±72.44	0.493	4.31±5.66	0.589
YES	54	87.41±46.36		3.83±3.24	
TNM					
T1	37	89.71±74.33	0.649	4.78±6.69	0.474
T2	46	97.44±60.04		4.00±3.53	
T3~T4	31	84.46±45.17		3.38±3.05	
ER					
Negative	40	85.10±58.67	0.409	3.79±3.55	0.629
Positive	74	95.09±62.86		4.24±5.17	
PR					
Negative	60	91.08±62.00	0.927	3.86±3.51	0.597
Positive	54	92.14±61.19		4.33±5.70	
HER-2					
Negative	74	95.38±65.82	0.371	3.95±3.53	0.825
Positive	40	84.56±52.13		4.15±5.19	
Age					
<49y	46	84.19±49.13	0.292	4.51±6.03	0.428
≥49y	68	96.59±68.29		3.72±3.57	
Tumor size					
<3cm	62	89.86±64.82	0.745	4.60±5.64	0.196
≥3cm	52	93.63±57.49		3.47±3.05	
Subtype					
Luminal A	17	107.84±82.80	0.558	5.57±8.79	0.392
Luminal B	58	90.73±55.11		3.79±3.44	
HER2-positive	20	78.74±50.50		3.15±2.90	
TNBC	19	93.16±67.94		4.62±4.11	

**Table 4:** Relationship between the absolute number of Bregs and its subset in PBMCs and the clinicopathological parameters of IBCa patients.

Variables	All cases	Bregs (×10^3^)	*P* value	PD-1^+^ Bregs (×10^3^)	*P* value
IBCa	114				
Grade					
G1	14	11.61±15.12	0.556	1.38±1.87	0.679
G2	73	8.95±6.73		1.11±1.48	
G3	27	9.70±8.21		0.95±1.15	
LN metastasis					
NO	60	10.03±9.94	0.446	1.19±1.77	0.512
YES	54	8.81±6.44		1.01±1.00	
TNM					
T1	37	9.87±10.32	0.702	1.36±1.84	0.443
T2	46	9.84±8.35		0.97±1.31	
T3~T4	31	8.33±5.89		1.01±1.11	
ER					
Negative	40	8.68±7.16	0.475	1.18±1.68	0.682
Positive	74	9.87±9.09		1.06±1.32	
PR					
Negative	60	9.47±7.33	0.983	1.16±1.52	0.655
Positive	54	9.44±9.62		1.04±1.38	
HER-2					
Negative	74	9.05±7.23	0.708	1.07±1.32	0.846
Positive	40	9.67±9.08		1.12±1.53	
Age					
<49y	46	9.41±7.04	0.962	1.20±1.80	0.547
≥49y	68	9.95±10.47		1.08±1.18	
Tumor size					
<3cm	62	9.40±8.69	0.944	1.19±1.54	0.500
≥3cm	52	9.51±8.24		1.00±1.35	
Subtype					
Luminal A	17	12.08±13.85	0.464	1.21±1.72	0.217
Luminal B	58	9.10±7.07		1.00±1.18	
HER2-positive	20	7.78±6.51		0.75±0.92	
TNBC	19	9.94±7.86		1.67±2.17	

**Figure 2:**
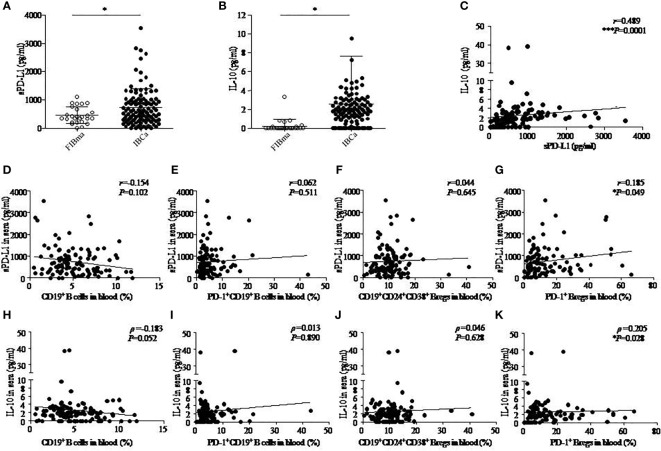
The correlation among IL-10, sPD-L1, and CD19^+^ B-cell subsets in IBCa. The production of soluble programmed death ligand 1 (sPD-L1) and interleukin-10 (IL-10) in the sera of FIBma (N = 26) and IBCa patients (N = 114) was measured by enzyme-linked immunosorbent assay (ELISA), as described in “Materials and methods”. The correlations between sPD-L1 and IL-10, sPD-L1 and CD19^+^ B-cell subsets, and also IL-10 and CD19^+^ B-cell subsets in IBCa patients (N=114) were investigated. sPD-L1 levels in the sera of IBCa patients and FIBma patients **(A)**. IL-10 levels in the sera of IBCa patients and FIBma patients **(B)**. The correlation between the levels of IL-10 and sPD-L1 in the sera from IBCa patients ***P = 0.0001 **(C)**. The correlation between the abundance of CD19^+^ B cells and sPD-L1 in the sera from IBCa **(D)**. The correlation between the abundance of PD-1^+^CD19^+^ B cells and sPD-L1 in the sera from IBCa **(E)**. The correlation between the abundance of CD19^+^CD24^+^CD38^+^ Bregs and sPD-L1 in the sera from IBCa **(F)**. The correlation between the abundance of PD-1^+^CD19^+^CD24^+^CD38^+^ Bregs and sPD-L1 in the sera from IBCa **(G)**. The correlation between the abundance of CD19^+^ B cells and IL-10 in the sera from IBCa **(H)**. The correlation between the abundance of PD-1^+^CD19^+^ B cells and IL-10 in the sera from IBCa **(I)**. The correlation between the abundance of CD19^+^CD24^+^CD38^+^ Bregs and IL-10 in the sera from IBCa **(J)**. The correlation between the abundance of PD-1^+^CD19^+^CD24^+^CD38^+^ Bregs and IL-10 in the sera from IBCa **(K)**. Each dot represents an individual patient. All values show the means ± SEM. Data were analyzed by unpaired Student’s *t*-test (A-B), Pearson’s correlation (C-G), and Spearman’s correlation (H-K). ns, not statistically significant, *P <0.05 and ***P <0.001.

As expected, the levels of sPD-L1 and IL-10 in the serum of patients with IBCa measured by ELISA were higher than those with FIBma (*P* = 0.029 and *P* = 0.023, respectively) (**Figures 2A, B** **)**. Further analysis of the relationships between the level of sPD-L1 or IL-10 and the histopathological characteristics of IBCa demonstrated that there were no significant associations with histological grade, lymph node metastasis, TNM stage, tumor size, ER status, or PR status (*P* > 0.05 for all; **Table 5**).

**Table 5:** Relationship between the level of IL-10 and sPD-L1 in serum and the clinicopathological parameters of IBCa patients.

Variables	All cases	IL-10 (pg/ml)	*P* value	sPD-L1(pg/ml)	*P* value
IBCa	114				
Grade					
G1	14	1.96±1.48	0.415	683.42±343.68	0.899
G2	73	2.25±4.47		738.59±648.50	
G3	27	3.69±7.43		780.63±755.17	
LN metastasis					
NO	60	2.58±5.14	0.955	751.64±647.10	0.864
YES	54	2.53±5.12		730.81±644.48	
TNM					
T1	37	3.08±6.26	0.243	894.22±683.60	0.177
T2	46	1.59±1.80		630.99±684.53	
T3~T4	31	3.37±6.63		724.20±496.28	
ER					
Negative	40	2.70±6.06	0.827	622.71±409.51	0.147
Positive	74	2.48±4.56		806.13±733.90	
PR					
Negative	60	2.23±5.00	0.478	706.60±615.37	0.540
Positive	54	2.92±5.25		780.87±676.18	
HER-2					
Negative	74	3.13±6.22	0.107	753.59±700.43	0.791
Positive	40	1.51±1.21		719.91±528.39	
Age					
<49y	46	2.57±5.82	0.985	641.36±581.45	0.171
≥49y	68	2.55±4.61		809.70±677.35	
Tumor size					
<3cm	62	2.52±4.93	0.927	812.61±633.00	0.200
≥3cm	52	2.61±5.36		657.32±650.90	
Subtype					
Luminal A	17	2.24±1.40	0.446	652.75±520.22	0.344
Luminal B	58	2.58±5.11		848.37±778.09	
HER2-positive	20	1.37±1.18		650.30±371.71	
TNBC	19	4.01±8.60		592.32±464.39	

Furthermore, we investigated the correlations between sPD-L1 and IL-10, sPD-L1 and CD19^+^ B-cell subsets, and also IL-10 and CD19^+^ B-cell subsets in IBCa patients. The results showed significantly positive correlations between the levels of sPD-L1 and IL-10 in the serum of IBCa patients (*r* = 0.489, *P* = 0.0001; **Figure 2C**), the percentage of PD-1^+^CD19^+^CD24^+^CD38^+^ B cells and sPD-L1 levels (*r* = 0.185, *P* = 0.049; **Figure 2G**), and the percentage of PD-1^+^CD19^+^CD24^+^CD38^+^ B cells and IL-10 levels (ρ = 0.205, *P* = 0.028; **Figure 1K**). No significant differences were found, although there was a negative correlation between the percentage of CD19^+^ B cells and IL-10 in the serum of IBCa patients (ρ = −0.183, *P* = 0.052; **Figure 2H**) and the percentage of CD19^+^ B cells and sPD-L1 in the serum of IBCa patients (*r* = −0.154, *P* = 0.102; **Figure 2D**). No correlations were observed between sPD-L1 levels and the percentage of PD-1^+^CD19^+^ B cells (**Figure 2E**), sPD-L1 and CD19^+^CD24^+^CD38^+^ B cells (**Figure 2F**), IL-10 and PD-1^+^ B cells (**Figure 2I**), or IL-10 and CD19^+^CD24^+^CD38^+^ B cells (**Figure 2J**).

### sPD-L1 contributed to the induction of CD19^+^CD24^+^CD38^+^ Bregs in an *in vitro* system

**Figure 3:**
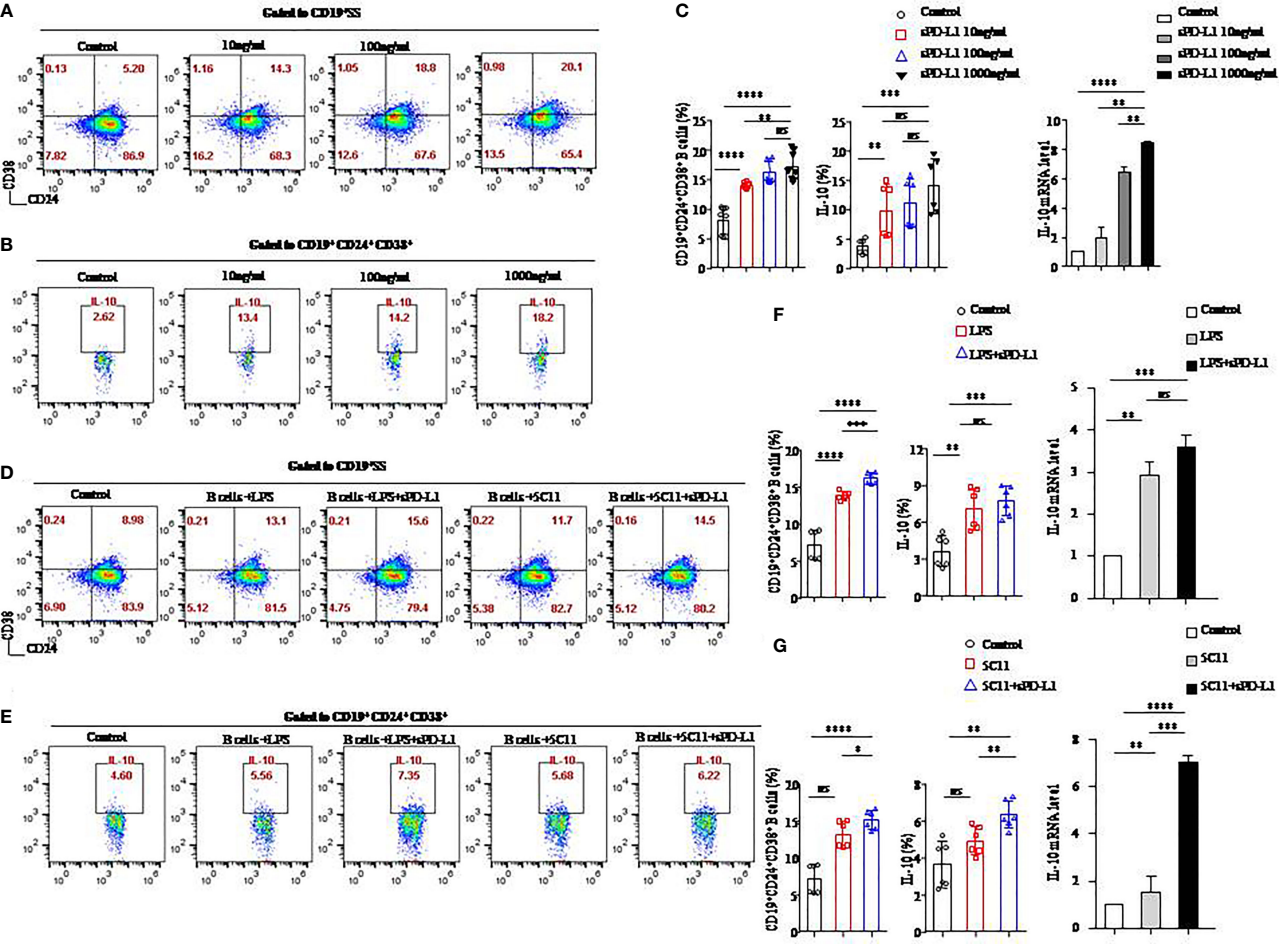
sPD-L1 mediated the differentiation of CD19^+^ B cells into CD19^+^CD24^+^CD38^+^ Bregs. CD19^+^ B cells enriched from the peripheral blood of healthy individuals were treated with sPD-L1 at doses from 10, 100, to 1,000 ng/ml. After 2 days, CD19^+^ B cells were collected and measured by flow cytometry. Representative flow cytometry dot plot of CD24 and CD38 expression in total CD19^+^ B cells **(A)** and IL-10 secreted by CD19^+^CD38^+^CD24^+^ B cells **(B)**. The proportion of CD19^+^CD38^+^CD24^+^ B cells (n = 6) and IL-10 secreted by CD19^+^CD38^+^CD24^+^ B cells (n = 6) was measured by flow cytometry **(C)**. The expression of IL-10 mRNA induced by sPD-L1 **(C)**. CD19^+^ B cells enriched from the peripheral blood of healthy individuals were activated with LPS, 5C11 (anti-CD40 Ab), or sPD-L1 individually or combinatorially, and flow cytometry dot plot of CD24 and CD38 expression in total CD19^+^ B cells **(D)** and IL-10 secreted by CD19^+^CD38^+^CD24^+^ B cells **(E)**. The proportion of CD19^+^CD38^+^CD24^+^ B cells (n = 6) and IL-10 secreted by CD19^+^CD38^+^CD24^+^ B cells (n = 6) was measured by flow cytometry, and the expression of IL-10 mRNA induced by LPS or sPD-L1 individually or combinatorially was measured **(F)**. The proportion of CD19^+^CD38^+^CD24^+^ B cells (n = 6) and IL-10 secreted by CD19^+^CD38^+^CD24^+^ B cells (n = 6) were measured by flow cytometry, and the expression of IL-10 mRNA was induced by 5C11 or sPD-L1 individually or combinatorially **(G)**. Data represent the means ± SEM of at least three independent experiments and were analyzed by Student’s *t* test. ns, not statistically significant. *P <0.05, **P <0.01, *** *P <*0.001, and ****P <0.0001.

To determine the role of the PD-1/PD-L1 axis on B-cells differentiation, we established a CD19^+^ B-cell incubation system *in vitro* induced by sPD-L1. We detected that the expression levels of CD24 and CD38 increased on total CD19^+^ B cells (**Figure 3A**) and that IL-10 secretion increased from CD19^+^CD24^+^CD38^+^ B cells (**Figure 3B**) in the incubation system. The expression levels of CD24 and CD38 on CD19^+^ B cells, IL-10 secretion by CD19^+^CD24^+^CD38^+^ B cells, and IL-10 mRNA levels in total CD19^+^ B cells in the incubation system were significantly increased compared to those in the control group in a dose-independent manner, as were the absolute numbers of CD19^+^CD24^+^CD38^+^ B cells and IL-10^+^ B cells (**Figure 3C**, **Supplemental Figure 2A**). The results indicated that the PD-1/PD-L1 axis may play an important role in CD19^+^CD24^+^CD38^+^ B-cell promotion and IL-10 secretion.

Because LPS and anti-CD40 antibody (5C11) are regarded as stimulators (20) of CD19^+^ B-cells activation, we further investigated the PD-1/PD-L1 axis on B cells activated by LPS or 5C11 combined with sPD-L1 and evaluated the percentage of CD19^+^CD24^+^CD38^+^ B cells (**Figure 3D**), IL-10 (**Figure 3E**) secreted by CD19^+^CD24^+^CD38^+^ B cells, and IL-10 mRNA levels (**Figures 3F, G** **)** in total CD19^+^ B cells. The percentage of CD19^+^CD24^+^CD38^+^ B cells, IL-10 secretion, and IL-10 mRNA levels was significantly increased compared to those in the control group in the LPS-activated incubation system with or without sPD-L1 (*P* < 0.05 for all; **Figure 3F**). Although the percentage and absolute number of CD19^+^CD24^+^CD38^+^ B cells were significantly different between the LPS group and the LPS combined with the sPD-L1 group (*P* = 0.0001; **Figure 3F**) (*P* = 0.004; **Supplemental Figure 2B**), the percentage of IL-10 secretion, IL-10 mRNA levels, and the absolute number of IL-10^+^ B cells was not significantly different between them (*P* = 0.426 and *P* = 0.199, respectively; **Figure 3F**) (*P* = 0.190; **Supplemental Figure 2B**). The highest levels of CD19^+^CD24^+^CD38^+^ B cells and IL-10 secretion and IL-10 mRNA were found in 5C11 combined with sPD-L1 compared to 5C11 alone or not (*P*<0.05 for all; **Figure 3G**). Although the percentage of IL-10 secretion was higher in 5C11 combined with the sPD-L1 group than in the 5C11 alone group (*P*=0.009, **Figure 3G**), the absolute number of CD19^+^CD24^+^CD38^+^ B cells and IL-10 secretion was not significantly different between the two groups (*P* = 0.807 and *P* = 0.899, respectively, **Supplemental Figure 2C**).

We speculated that sPD-L1 in the serum of IBCa patients will lead to CD19^+^CD24^+^CD38^+^ B-cell promotion and IL-10 secretion and that CD40 signaling might enhance the effects on B-cell differentiation.

### CD19^+^ B cells contributed to the induction of T_regs_ in an *in vitro* system by sPD-L1

**Figure 4:**
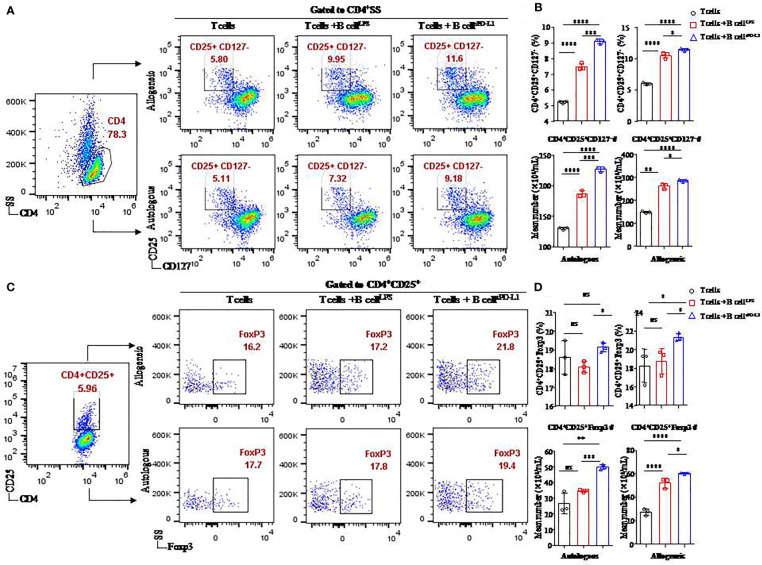
Function of CD19^+^ B cells stimulated by sPD-L1 in a T/B lymphocyte coculture system. Representative flow cytometry dot plot of CD25^+^CD127^−^ expression in total CD4^+^ T cells **(A)** and Foxp3 expression in CD4^+^ CD25^+^ T cells **(C)** from autologous or allogeneic healthy individuals cocultured with CD19^+^ B cells pretreated with LPS or sPD-L1. The proportion and the absolute number of CD4^+^CD25^+^CD127^−^ T cells (n = 3) were measured by flow cytometry **(B)**, and the proportion and the absolute number of Foxp3 expression in CD4^+^CD25^+^ T cells (n = 3) were measured by flow cytometry **(D)** in the B/T lymphocyte coculture system. Data represent the means ± SEM of at least three independent experiments and were analyzed by Student’s *t*-test. ns, not statistically significant. *P <0.05, **P <0.01, ***P <0.001, and ****P <0.0001.

To investigate whether sPD-L1 influences the development of T_regs_, we cultured CD19^+^ B cells stimulated by LPS or sPD-L1 with CD4^+^ T cells collected from allogeneic or autologous healthy individuals. We detected the percentages of CD4^+^CD25^+^CD127^−^ T cells (**Figure 4A**) and Foxp3 on CD4^+^CD25^+^ T cells (**Figure 4C**) in the coincubation system.

Higher levels of CD4^+^CD25^+^CD127^−^ T cells (11.43 ± 0.21% or 9.08 ± 0.15%, respectively) were found in CD4^+^ T cells originating from either allogeneic or autologous healthy individuals cultured with CD19^+^ B cells pretreated with sPD-L1 (CD19^+^ B-cell^sPD-L1^) compared to the CD19^+^ B-cell^LPS^ group (10.48 ± 0.46% or 7.47 ± 0.22%, respectively) (*P* = 0.0005, *P* = 0.032, respectively; **Figure 4B**) and the control group (5.97 ± 0.19% or 5.21 ± 0.09%, respectively) (*P* < 0.0001, *P* < 0.0001, respectively; **Figure 4B**). Meanwhile, the absolute number of CD4^+^CD25^+^CD127^−^ T cells was highest in the CD19^+^ B-cell^sPD-L1^ group among the coincubation groups regardless of whether CD4^+^ T cells were from allogeneic or autologous healthy individuals (*P* < 0.05 for all; **Figure 4B**).

Foxp3, a transcription factor of T_regs_, was detected in the coincubation system (**Figure 4C** **)**. The results indicated that the expression of Foxp3 was higher in the CD19^+^ B-cell^sPD-L1^ group, in which CD4^+^ T cells were obtained from allogeneic individuals, than in the CD19^+^ B-cell^LPS^ group (23.33 ± 0.42% *vs*. 18.77 ± 1.37%, *P* = 0.036; **Figure 4D**) or the control group (23.33 ± 0.42% *vs*. 18.27 ± 1.79%, *P* = 0.045; **Figure 4D**). Similarly, the absolute number of CD4^+^CD25^+^ Foxp3^+^ T cells was also highest in the CD19^+^ B-cell^sPD-L1^ group among the three groups (**Figure 4D**).

We observed that the expression of Foxp3 on CD4^+^CD25^+^ T cells in the CD19^+^ B-cell^sPD-L1^ group was higher than that in the CD19^+^ B-cell^LPS^ group with CD4^+^ T cells originating from autologous individuals (19.13 ± 0.25% vs. 18.10 ± 0.30%, *P* = 0.010; **Figure 4D**), but no significant difference was observed between the CD19^+^ B-cell^sPD-L1^ group and the control group (19.13 ± 0.25% vs. 18.60 ± 0.90%, *P* = 0.379; **Figure 4D**). Moreover, the absolute number of CD4^+^CD25^+^Foxp3^+^ T cells in the CD19^+^ B-cell^sPD-L1^ group was significantly higher than those in the CD19^+^ B-cell^LPS^ and control groups (*P* = 0.032, *P* < 0.0001, respectively) (**Figure 4D**).

Together, these results suggested that sPD-L1 played a critical role in regulating T_regs_ induction *via* CD19^+^ B cells.

### The soluble form of PD-L1 originating from the PD-L1^hi^ breast cancer cell line mediates the differentiation of CD19^+^ B cells in B-cell/tumor coculture systems

**Figure 5:**
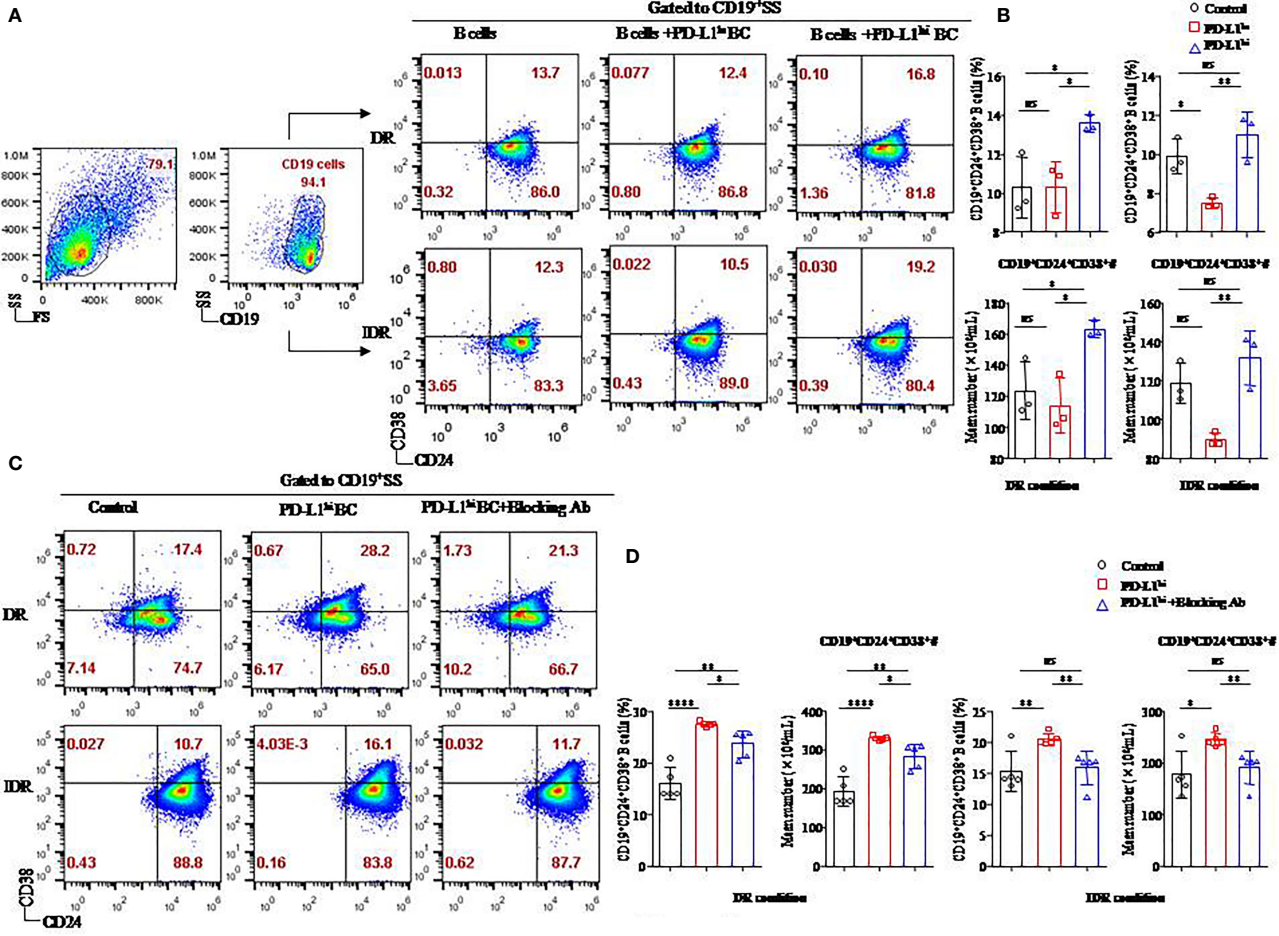
The soluble form of PD-L1 originating from a breast cancer cell line induced CD19^+^CD24^+^CD38^+^ Bregs in coculture systems. Representative flow cytometry dot plot of CD24 and CD38 expression in total CD19^+^ B cells cultured with PD-L1^hi^ MDA-MB231 cells or PD-L1^lo^ MCF-7 cells in DR or IDR conditions **(A)**. The percentages and the absolute number of CD19^+^CD38^+^CD24^+^ B cells were detected in the culture systems with PD-L1^hi^ MDA-MB231 cells or PD-L1^lo^ MCF-7 cells in DR (n = 3) or IDR (n = 3) conditions **(B)**. Representative flow cytometry dot plot of CD24 and CD38 expression in total CD19^+^ B cells cultured with PD-L1^hi^ MDA-MB231 with or without PD-L1–blocking antibody treatment in DR or IDR conditions **(C)**. The percentages and the absolute number of CD19^+^CD38^+^CD24^+^ B cells were detected in DR (n = 5) or IDR (n = 5) conditions with PD-L1^hi^ MDA-MB231 cells pretreated with or without PD-L1–blocking antibody **(D)**. Data represent the means ± SEM of at least three independent experiments and were analyzed by Student’s *t*-test. ns, not statistically significant. *P <0.05, **P <0.01, and ****P <0.0001.

As in our previous studies (5), MDA-MB231 (PD-L1^hi^ BC), a TNBC breast cancer cell line, expressed high level of PD-L1 (**Supplemental Figure 1A**). More importantly, the soluble form of PD-L1 on PD-L1^hi^ BC was reported in our previous research (21). To investigate the function of sPD-L1 from MDA-MB231 on the differentiation of CD19^+^ B cells, we established MDA-MB231 cells incubated with CD19^+^ B cells activated by LPS in DR or IDR conditions compared to PD-L1–deficient MCF-7 breast cancer cells (PD-L1^lo^ BC) (**Supplemental Figure 1A**) (**Figure 5A**).

Similar to our previous studies, both a higher percentage and absolute number of CD19^+^CD24^+^CD38^+^ B cells were found in the CD19^+^ B cells cultured with PD-L1^hi^ BC cells than in the CD19^+^ B cells cultured with PD-L1^lo^ BC cells or control cells under DR conditions (*P* < 0.05 for all; **Figure 5B**). The average frequency and absolute number of CD19^+^CD24^+^CD38^+^ B cells in the PD-L1^hi^ BC group were higher than those in the PD-L1^lo^ BC group under IDR conditions (*P* = 0.008, *P* = 0.007, respectively; **Figure 5B**). Although the percentage and absolute number of CD19^+^CD24^+^CD38^+^ B cells in the PD-L1^hi^ BC group were higher than those in the control group under IDR culture condition, no significant difference was observed between the PD-L1^hi^ BC group and the control group (*P* = 0.268 and *P* = 0.269, respectively; **Figure 5B**).

To confirm whether the induction of CD19^+^CD24^+^CD38^+^ Bregs was due to PD-L1, we blocked the expression of PD-L1 in MDA-MB231 cells by using a specific anti–PD-L1 blocking antibody (**Supplemental Figure 1B**). PD-L1^hi^ MDA-MB231 cells with PD-L1 blockade were then cocultured with activated CD19^+^ B cells under DR or IDR conditions (**Figure 5C**). The percentage and absolute number of CD19^+^CD24^+^CD38^+^ Bregs were reduced in the PD-L1^hi^ BC blockade group compared to the PD-L1^hi^ BC and control groups under DR or IDR culture conditions (*P* < 0.05 for all; **Figure 5D**).

These results demonstrated that sPD-L1 from MDA-MB231 cells mediated the differentiation of CD19^+^ B cells in the coculture system, which could be reduced by an anti-human PD-L1 blocking antibody.

### Apoptosis of breast cancer cells contributed to PD-L1 signal blockade in a T/B lymphocyte/tumor cell coculture system

**Figure 6:**
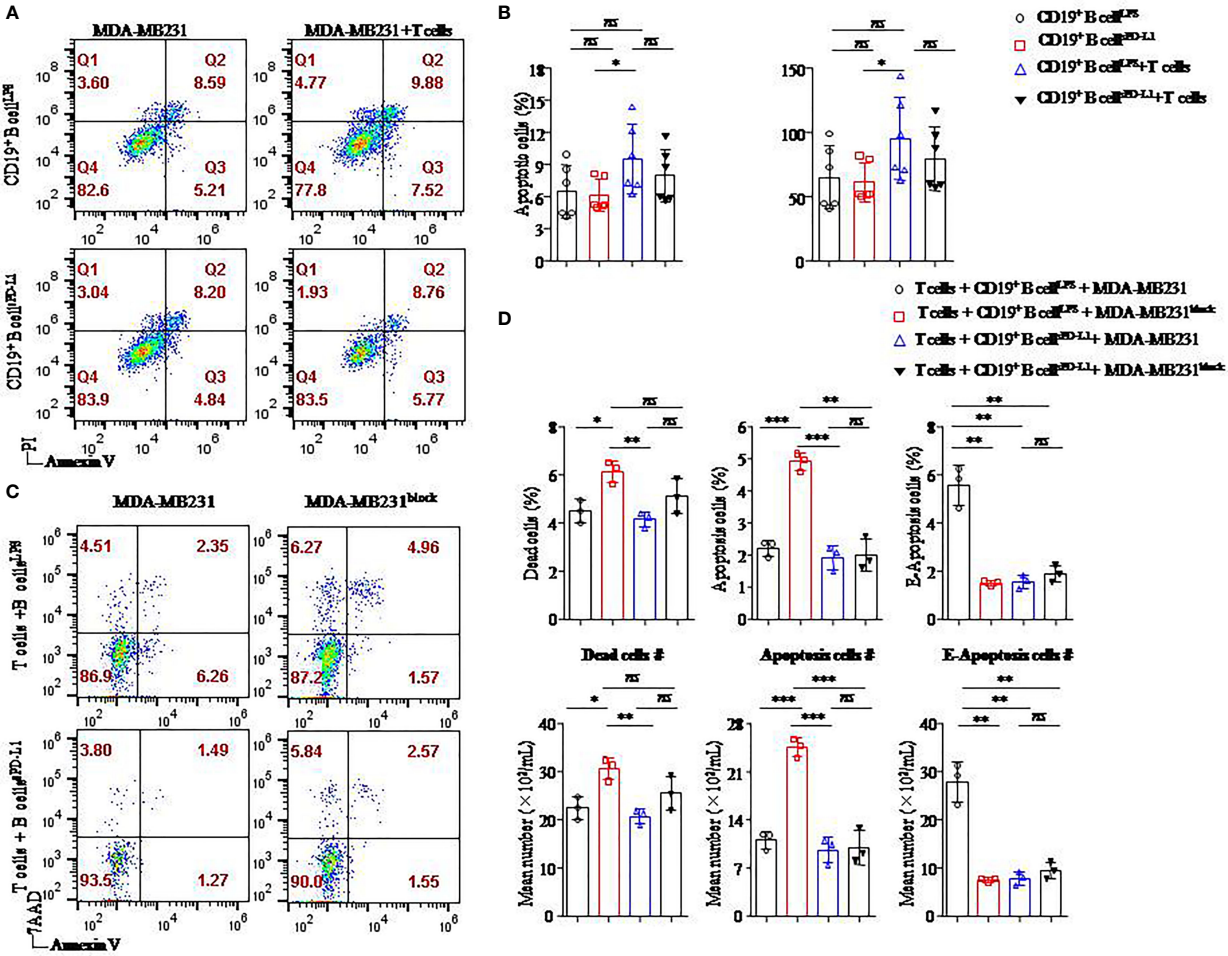
Breast cancer cell apoptosis was induced when the PD-L1 signal was blocked. Representative flow cytometry dot plot of apoptosis of PD-L1^hi^ MDA-MB231 cells cultured with CD4^+^ T cells and CD19^+^ B cells pretreated with LPS or sPD-L1 **(A)**. The percentage and absolute number of apoptotic cells were detected in PD-L1^hi^ MDA-MB231 cells (n = 6) cultured with CD19^+^ B cells pretreated with LPS or sPD-L1 with or without CD4^+^ T cells **(B)**. Representative flow cytometry dot plot of death, apoptosis, and E-apoptosis of PD-L1^hi^ MDA-MB231 cells with or without PD-L1–blocking antibody treatment in the culture system including CD4^+^ T cells and B cells pretreated with LPS or sPD-L1 **(C)**. The percentage and the absolute number of dead, apoptotic, and E-apoptotic cells were detected in PD-L1^hi^ MDA-MB231 cells (n = 3) cultured with CD4^+^ T cells and CD19^+^ B cells pretreated with LPS or sPD-L1 with or without PD-L1–blocking antibody treatment **(D)**. Data represent means ± SEM of at least three independent experiments and were analyzed by unpaired Student’s *t*-test. ns, not statistically significant. *P <0.05, **P <0.01, and ***P <0.001.

In our present study, sPD-L1 played an important role in mediating the differentiation of CD19^+^ B cells and promotion of T_regs_. We analyzed the influence of sPD-L1 on the survival of PD-L1^hi^ MDA-MB231 in a T-/B-cell/tumor coculture system with CD19^+^ B cells activated by LPS or sPD-L1 (**Figure 6A**) and further investigated PD-L1 signal blockade for immunotherapy in PD-L1^hi^ MDA-MB231, a breast cancer cell line of TNBC (**Figure 6C**).

The highest percentage and absolute number of apoptotic cells were detected on MB-MDA231 cells in T-/B-cell/tumor coculture systems in which CD19^+^ B cells were stimulated by LPS among the other groups, and the percentages of apoptotic cells were decreased in the coculture experiment in which CD19^+^ B cells were stimulated by sPD-L1 (**Figure 6B**). Whether CD19^+^ B cells were pretreated with LPS or sPD-L1, the percentage and absolute number of apoptotic cells were not significantly different in the coculture system without CD4^+^ T cells (*P* > 0.05 for all; **Figure 6B**). This result indicated that CD19^+^ B cells mediated the antitumor immune response *via* CD4^+^ T cells.

Furthermore, we detected the death of MB-MDA231 cells in the T-/B-cell coculture system, whereas the PD-L1 signal was blocked by a specific antibody or not (**Figure 6C**). Compared to CD19^+^ B cells stimulated by LPS or sPD-L1 alone, the percentage and absolute numbers of dead and apoptotic cells increased significantly in the group in which CD19^+^ B cells were stimulated by LPS with PD-L1 signal blockade (*P* < 0.05 for all; **Figure 6D**). Meanwhile, the percentages of dead, apoptotic, and early apoptotic cells were the lowest in the group with CD19^+^ B cells stimulated by sPD-L1 and recruited when the PD-L1 signal was blocked by the anti–PD-L1 antibody, but no significant difference was detected (*P* > 0.05 for all; **Figure 6D**). Moreover, the percentage of early apoptotic cells was the highest in the group in which CD19^+^ B cells were stimulated by LPS compared to the other groups (*P* < 0.05 for all; **Figure 6D**).

We speculated that CD19^+^ B cells stimulated by LPS played an important role in the antitumor immunity of CD4^+^ T cells and that PD-L1 signal blockade might enhance the effects on PD-L1^hi^ BC.

## Discussion

Bregs are a novel subset of B cells derived from various B-cell progenitors (5, 22). They show a characteristic expression pattern of CD24, CD38, CD5, CD1d, CD27, and PD-L1 (5, 23–25). Indeed, they can negatively regulate T-cell immune responses to maintain self-tolerance and prevent autoimmunity by secreting anti-inflammatory mediators such as IL-10, IL-35, and transforming growth factor β (TGF-β) (24). IL-10 production has been identified for the function of Bregs (24). In our previous studies, we identified a high percentage of CD19^+^CD24^+^CD38^+^ Bregs in the tumor microenvironment (TME) and peripheral blood of IBCa patients (5, 23). More importantly, we found that the percentage of CD19^+^CD24^+^CD38^+^ Bregs with high levels of IL-10 secretion was positively correlated with T_regs_ in the peripheral blood of IBCa patients (23).

In the present study, we found that PD-1 expression on CD19^+^CD24^+^CD38^+^ Bregs in PBMCs of IBCa patients was higher than that of FIBma patients. Moreover, a higher level of sPD-L1, a soluble form of PD-L1, was found in the serum of IBCa patients compared with FIBma patients. Additionally, a tight positive correlation was found between serum levels of sPD-L1 and IL-10, as well as between sPD-L1 levels and the percentage of PD-1^+^CD19^+^CD24^+^CD38^+^ Bregs among PBMCs in IBCa patients. PD-1^hi^ Bregs mediated the reduction and dysfunction of CD8^+^ T cells in the human hepatocellular carcinoma (26).

Based on the positive correlation between sPD-L1 level and the percentage of PD-1^+^CD19^+^CD24^+^CD38^+^ Bregs, we further investigated the function of sPD-L1 in the serum of breast cancer patients. Although several functions of sPD-L1 in the TME of advanced pancreatic cancer, hepatocellular carcinoma, and breast cancer have been explored (16, 17), the involvement of sPD-L1 in B-cell differentiation has not been clarified, except for several previous studies showing that sPD-L1 is associated with prognostic prediction and responses to immunotherapies (15, 17). Our findings confirmed that the percentage of CD19^+^CD24^+^CD38^+^ Bregs was markedly increased in a dose-dependent manner by sPD-L1 induction compared with the control. In addition, sPD-L1 treatment also dose-dependently enhanced the secretion of IL-10 by Bregs. Furthermore, we demonstrated that the soluble form of PD-L1, which was found in PD-L1^hi^ MDA-MB231 cells in our previous research (21), could increase the proportion of CD19^+^CD24^+^CD38^+^ Bregs. This effect of sPD-L1 could be inhibited by a specific PD-L1–blocking antibody (Ab). Based on these findings, we hypothesize that sPD-L1 from breast cancer cells actively promotes the differentiation of B cells into Bregs and suppresses antitumor activity both the local TME and the circulation of IBCa patients.

PD-L1, a 40-kDa type I transmembrane protein belonging to the B7 family, is expressed on the surfaces of both immune cells and non-immune cells, such as T cells, B cells, dendritic cells (DCs), macrophages, T_regs_, and several types of tumors cells (8). PD-1, a receptor of PD-L1, is an Ig superfamily member expressed on activated T cells and B cells, among others (11). Both PD-L1 and PD-1 are regarded as immune checkpoints, and the interaction between them can inhibit both proliferation and cytokine production by T_effs_ (9).

Wang et al. previously identified a new subset of B cells expressing PD-1 that could inhibit the functions of CD4^+^ T cells and CD8^+^ T cells in differentiated thyroid cancer (26). Xiao et al. demonstrated that this subset of PD-1^+^ B cells could significantly suppress the response of tumor-specific T cells and enhanced tumor growth *via* IL-10 in advanced hepatocellular carcinoma (22). In our previous studies, we also observed elevated percentages of CD4^+^CD25^+^CD127^−^ T_regs_ in the peripheral blood of IBCa patients compared with FIBma patients or healthy individuals and a positive correlation between T_regs_ and CD19^+^CD24^+^CD38^+^ Bregs in IBCa patients. Furthermore, we found that PD-L1 exhibited high expression on Bregs in IBCa patients and that its expression was positively correlated with the proportion of T_regs_ but negatively correlated with that of PD-1^hi^ T_effs_ (23).

T_regs_, a subset of CD4^+^ T cells, stably express Foxp3 and CD25, but exhibit no CD127 expression (27). CD4^+^CD25^+^CD127^−^ T_regs_ or CD4^+^CD25^+^ Foxp3^+^ T_regs_ can inhibit T_eff_ activation and induce T_eff_ apoptosis to maintain peripheral tolerance and immune homeostasis (28, 29). Several studies have also revealed that T_regs_ play critical roles in regulating antitumor immune suppression and tumor immune escape, metastasis, and relapse (28, 30). In this study, we further investigated and confirmed the immunosuppressive functions of Bregs derived from CD19^+^ B cells stimulated by sPD-L1 on CD4^+^ T-cell polarization and differentiation *in vitro*. We observed that CD19^+^ B cells pretreated with sPD-L1 could transformed CD4^+^ T cells originating from allogeneic or autologous sources into CD4^+^CD25^+^CD127^−^ T_regs_. Meanwhile, Foxp3, a transcription factor expressed specifically in T_regs_, was upregulated by sPD-L1 stimulation in the experiments. Tadmor et al. also observed increased T_regs_ expansion in the spleen, tumor-draining lymph nodes, and tumor beds of wild-type murine tumor model compared with their B-cell–deficient counterparts (31). Flores-Borja and colleagues confirmed that depletion of Bregs favored the activation of T cells and promoted their effector function (32).

**Figure 7:**
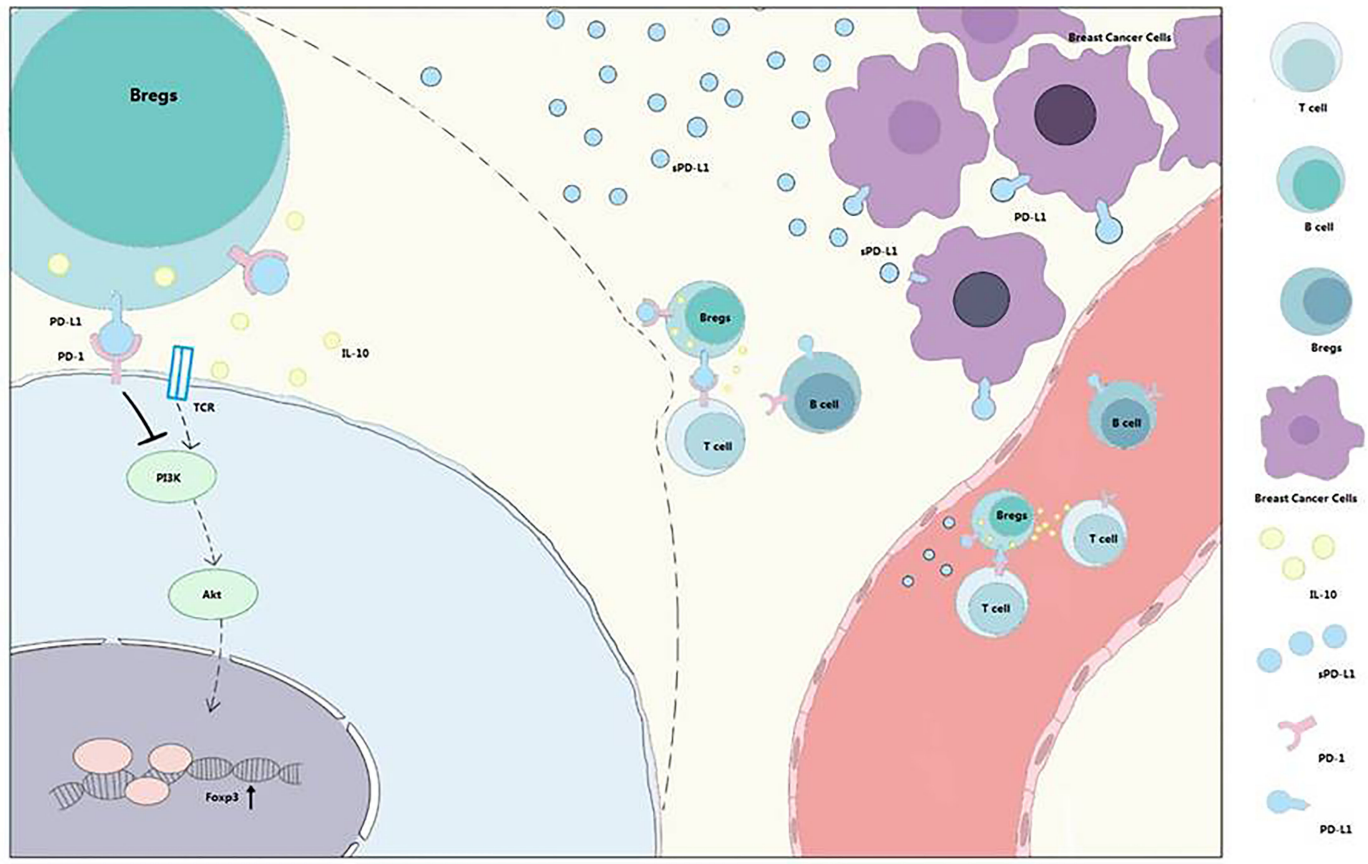
PD-1/PD-L1 signaling in Bregs differentiation and function. Bregs differentiation occurs in the TME or immune system of patients with breast cancer. sPD-L1 from breast cancer could upregulate the expression of CD24 and CD38 on CD19^+^ B cells and promote IL-10 secretion from CD24^+^CD38^+^CD19^+^ Bregs. With the interaction of B cells and T cells, PD-1/PD-L1 signaling contributes to T_regs_ promotion and effector T-cell (Teff) exhaustion. Meanwhile, sPD-L1 is also released into blood to further interact with CD19^+^ B cells and promotes differentiation and promotion of Bregs and T_regs_.

Bregs could induce T_regs_ *in vitro* (5), and the tumor-evoked Bregs could promote lung metastases in a mouse 4T1 model (33). Lee-Chang et al. revealed local intratumoral depletion of Bregs resulted in improved survival in a murine tumor model (34). PD-L1 signaling can also maintain the expression of Foxp3 during T_reg_ development (33). Based on these finding, we hypothesized that sPD-L1 from breast cancer could increase the proportion of Bregs and IL-10 secretion and induce T_reg_ differentiation and T_eff_ exhaustion (**Figure 7**).

Recently, Gallego-Valle et al. reported that combinatory CD40 ligand (CD40L) and bacteria-derived oligodeoxynucleotides (CpG-ODNs) could significantly induce the differentiation of B cells into functional Breg-like cells (35). Similarly, we observed that the proportion and the absolute number of CD19^+^CD24^+^CD38^+^ Bregs were increased in cells stimulated with CD40 agonist Abs and LPS alone or in combination with sPD-L1.

Therefore, we performed activated CD4^+^ T-cell/CD19^+^ B-cell/tumor cell coculture experiments to investigate the potential function of Bregs or sPD-L1 for immunotherapy. We observed higher percentages and absolute numbers of apoptotic MB-MDA231 cells in the coculture systems in which CD19^+^ B cells were activated and reduced percentages of apoptotic breast cancer cells in the coculture systems in which the CD19^+^ B cells were pretreated with sPD-L1. Furthermore, our finding demonstrated that the percentages and absolute numbers of dead and apoptotic PD-L1^hi^ MDA-MB231 cells increased significantly when the PD-L1 signal was blocked by antagonist antibody in the coculture system.

CD40 is a key costimulatory molecule belonging to the tumor necrosis factor receptor (TNFR) superfamily expressed on the surfaces of B cells, DCs, macrophages, and non-immune cells (36). The ligand for CD40 is CD40L, which is a member of the TNF superfamily (TNFSF) (37). CD40L is expressed on a variety of activated cell types, including activated CD4^+^ T cells, activated natural killer cells, memory CD8^+^ T cells, and activated platelets (36, 37). The CD40/CD40L interaction between T cells and B cells is necessary for T-cell activation, as well as for activation, differentiation, and proliferation of B cells (36). Together, these studies demonstrate the prospect of using a CD40 agonist strategy in patients for cancer therapy (36). Nonetheless, the proportion and function of Bregs induced by CD40 agonist alone or combining with sPD-L1 in antitumor action cannot be ignored. Therefore, treatment combining CD40 agonist and PD-L1 or PD-1 antagonist Abs might be more efficient and favor antitumor immunity (38, 39).

Breast cancer is the most commonly diagnosed cancer in females, and the survival rate of breast cancer patients has been increased over the past decade for targeted therapies (1), including tamoxifen for patients with luminal A and luminal B subtypes of IBCa (40) and trastuzumab for HER2-positive IBCa patients (41, 42). However, there are currently no effective therapeutic targets for TNBC patients (negative for ER, PR, and HER2) (1). In the present study, we observed that the percentages of Bregs and PD-1^+^ Bregs in TNBC patients were the highest among patients with different breast cancer subtypes. Furthermore, we confirmed that sPD-L1 alone or combined with LPS or CD40 agonist Ab could induce Bregs expansion to inhibit the antitumor response. Our study reveals that the levels of sPD-L1 or IL-10 in the serum or PD-1^+^ Bregs in the PBMCs of breast cancer patients might be predictors for immunotherapy, and combination PD-L1 antagonist and CD40 agonist Abs might be a more efficient immunotherapy for TNBC.

## Data availability statement

Publicly available datasets were analyzed in this study. This data can be found here: https://www.ncbi.nlm.nih.gov/nuccore/NM_000572.3 and https://www.ncbi.nlm.nih.gov/nucleotide/NM_001101.5?report=genbank&;log$=nuclalign&blast_rank=2&RID=X2V0UP33013.

## Ethics statement

The studies involving human participants were reviewed and approved by the ethics committee of the medical college of Soochow University, approval number ECSU-201600011. The patients/participants provided their written informed consent to participate in this study.

## Author contributions

XL, HD, SZ, QW, and FX designed and performed most of the *in vitro* experiments. XL, QW, and FX wrote the manuscript. XL, HD, SZ, and LP performed the experiments. SZ, WL, ZW, JL, and YW collected samples and performed experiments. XL, HD, QQ, SW, YY, and FX analyzed all of the data. QW and FX designed and analyzed the study. All authors contributed to the article and approved the submitted version.

## Funding

This study was funded in part by the National Natural Science Foundation of China (NSFC 81372343, 81772809 and 82073374), the Key University Science Research Project of Jiangsu Province (20KJA180002), and the Priority Academic Program Development of Jiangsu Higher Education Institutions (PAPD).

## Conflict of interest

The authors declare that the research was conducted in the absence of any commercial or financial relationships that could be construed as a potential conflict of interest.

## Publisher’s note

All claims expressed in this article are solely those of the authors and do not necessarily represent those of their affiliated organizations, or those of the publisher, the editors and the reviewers. Any product that may be evaluated in this article, or claim that may be made by its manufacturer, is not guaranteed or endorsed by the publisher.

## Supplementary material

Supplementary Figure 1 The pattern of PD-L1 in breast cancer and its inhibition by specific antibodies. The expression of PD-L1 and PD-1 in breast cancer cell lines was analyzed by flow cytometry. MDA-MB231 cells expressed high levels of PD-L1 (99.1%) compared with MCF-7 cells (11.3%) **(A)**. To confirm the relationship between PD-L1 and CD19^+^ B cells, a PD-L1-blocking antibody was used to block the expression of PD-L1 at different dilutions **(B)**. Data represent the mean and SEM of at least three independent experiments.

Supplementary Figure 2 The absolute number of CD19^+^CD24^+^CD38^+^ B cells and IL-10^+^ B cells induced by sPD-L1. CD19^+^ B cells enriched from the peripheral blood of healthy individuals were treated with sPD-L1 at doses from 10 ng/mL, 100 ng/mL to 1000 ng/mL. After 2 days, CD19^+^ B cells were collected, and the absolute numbers of CD19^+^CD38^+^CD24^+^ B cells (n=6) and IL-10^+^ B cells on CD19^+^CD38^+^CD24^+^ B cells (n=6) were measured by flow cytometry (A). The absolute numbers of CD19^+^CD38^+^CD24^+^ B cells (n=6) and IL-10^+^ B cells on CD19^+^CD38^+^CD24^+^ B cells (n=6) were measured by flow cytometry induced by LPS or sPD-L1 individually or combinatorially (B). The absolute numbers of CD19^+^CD38^+^CD24^+^ B cells (n=6) and IL-10^+^ B cells on CD19^+^CD38^+^CD24^+^ B cells (n=6) were measured by flow cytometry induced by 5C11 or sPD-L1 individually or combinatorially (C). Data represent the mean ± SEM of at least three independent experiments and were analyzed by Student’s *t* test. ns, not statistically significant. **P <*0.05, ***P <*0.01, ****P <*0.001, and *****P <*0.0001.
