# Supplementary figures and images for: The interaction between the soluble programmed death ligand-1 (sPD-L1) and PD-1+ regulator B cells mediates immunosuppression in triple-negative breast cancer
Source: Front Immunol. 2022 Jul 22;13:830606. doi: 10.3389/fimmu.2022.830606 (PMC9354578; doi:10.3389/fimmu.2022.830606)

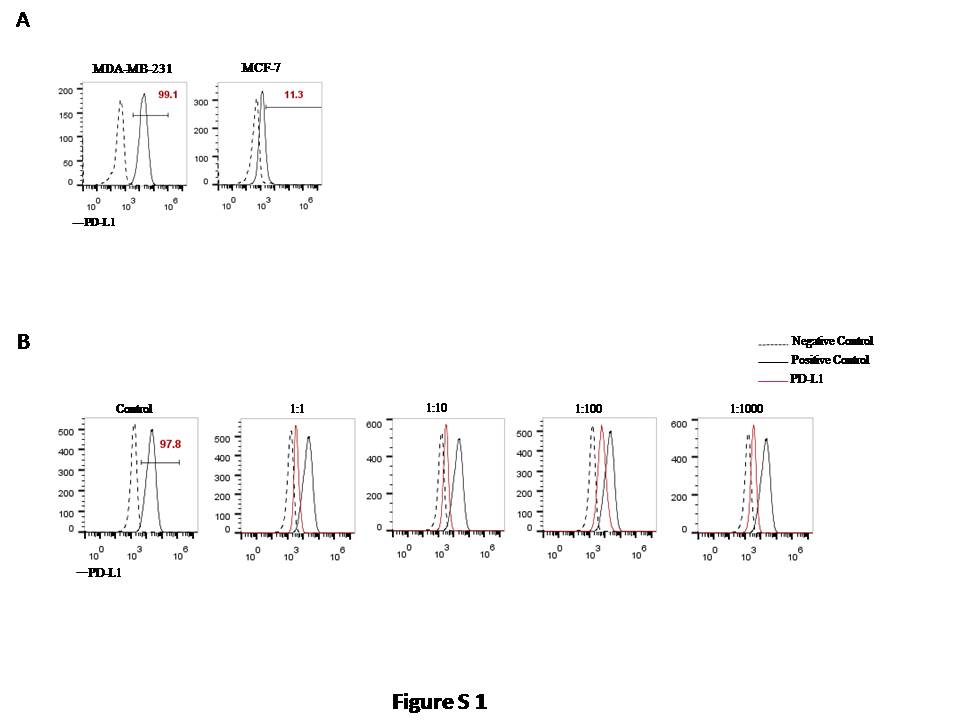

Supplement: Supplementary Figure 1 — The pattern of PD-L1 in breast cancer and its inhibition by specific antibodies. The expression of PD-L1 and PD-1 in breast cancer cell lines was analyzed by flow cytometry. MDA-MB231 cells expressed high levels of PD-L1 (99.1%) compared with MCF-7 cells (11.3%) (A). To confirm the relationship between PD-L1 and CD19+ B cells, a PD-L1-blocking antibody was used to block the expression of PD-L1 at different dilutions (B). Data represent the mean and SEM of at least three independent experiments. [file Image_1.jpeg]

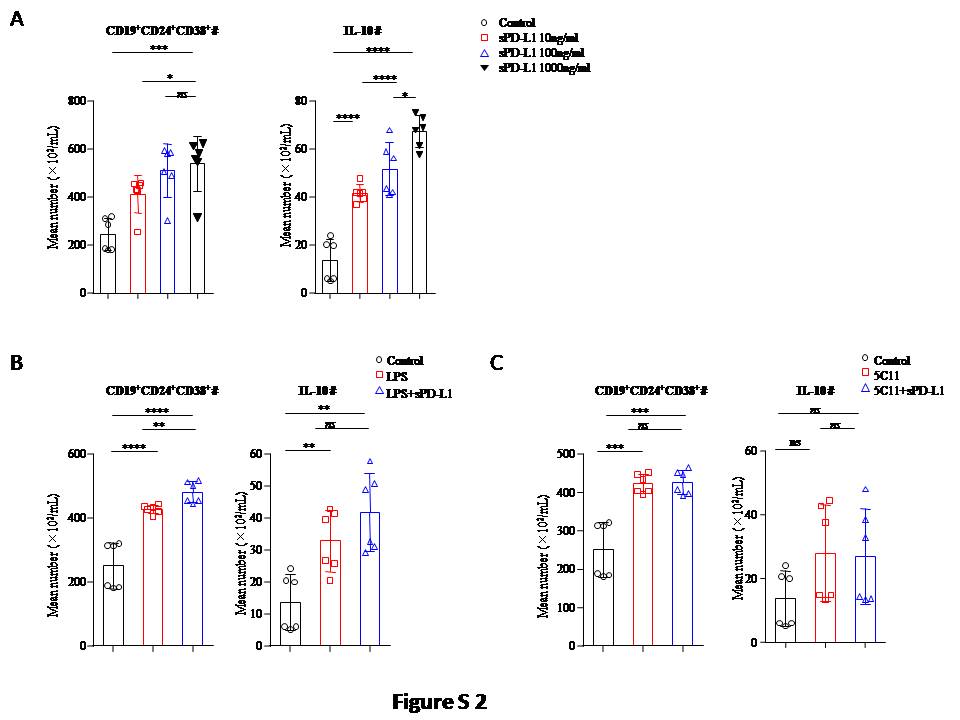

Supplement: Supplementary Figure 2 — The absolute number of CD19+CD24+CD38+ B cells and IL-10+ B cells induced by sPD-L1. CD19+ B cells enriched from the peripheral blood of healthy individuals were treated with sPD-L1 at doses from 10 ng/mL, 100 ng/mL to 1000 ng/mL. After 2 days, CD19+ B cells were collected, and the absolute numbers of CD19+CD38+CD24+ B cells (n=6) and IL-10+ B cells on CD19+CD38+CD24+ B cells (n=6) were measured by flow cytometry (A). The absolute numbers of CD19+CD38+CD24+ B cells (n=6) and IL-10+ B cells on CD19+CD38+CD24+ B cells (n=6) were measured by flow cytometry induced by LPS or sPD-L1 individually or combinatorially (B). The absolute numbers of CD19+CD38+CD24+ B cells (n=6) and IL-10+ B cells on CD19+CD38+CD24+ B cells (n=6) were measured by flow cytometry induced by 5C11 or sPD-L1 individually or combinatorially (C). Data represent the mean ± SEM of at least three independent experiments and were analyzed by Student’s t test. ns, not statistically significant. *P <0.05, **P <0.01, ***P <0.001, and ****P <0.0001. [file Image_2.jpeg]
