# Supplementary material for: The interaction between the soluble programmed death ligand-1 (sPD-L1) and PD-1+ regulator B cells mediates immunosuppression in triple-negative breast cancer
Source: Front Immunol. 2022 Jul 22;13:830606. doi: 10.3389/fimmu.2022.830606 (PMC9354578; doi:10.3389/fimmu.2022.830606)
Supplement: Supplementary file 3 [file Table_1.docx]

**Supplementary Table 1. Clinical characteristic of IBCa patients**

| Variables |  | No. of patients |
| --- | --- | --- |
| T Status | <3cm | 62 |
|  | ≥3cm | 52 |
| N Status | No | 60 |
|  | N1/N2/N3 | 54 |
| TNM | I | 36 |
|  | II | 48 |
|  | III | 19 |
|  | IV | 11 |
| Grade | I | 14 |
|  | II | 73 |
|  | III | 27 |
| ER | - | 40 |
|  | + | 64 |
| PR | - | 60 |
|  | + | 54 |
| HER2 | - | 74 |
|  | + | 40 |
| Molecular Subtype | Luminal A | 17 |
|  | Luminal B | 58 |
|  | HER2 over-expression | 20 |
|  | TNBC | 19 |

Summary of the clinical characteristic of IBCa patients. In brief,
