# Supplementary material for: The interaction between the soluble programmed death ligand-1 (sPD-L1) and PD-1+ regulator B cells mediates immunosuppression in triple-negative breast cancer
Source: Front Immunol. 2022 Jul 22;13:830606. doi: 10.3389/fimmu.2022.830606 (PMC9354578; doi:10.3389/fimmu.2022.830606)
Supplement: Supplementary file 4 [file Table_2.docx]

**Supplementary Table 2. The sequence of mRNA for β-actin and IL-10**

| Name | Primer sequence |  |
| --- | --- | --- |
| IL-10-Homo-mRNA1 | Sense  Antisense | 5’- AAG ACC CAG ACA TCA AGG CG -3’  5’- AAT CGA TGA CAG CGC CGT AG -3’ |
| β-actin -Homo- mRNA | Sense  Antisense | 5’- ATT GGC AAT GAG CGG TTC -3’  5’- GGA TGC CAC AGG ACT CCAT -3’ |

The sequence of mRNA
